# Deletion of *BSG1* in *Chlamydomonas reinhardtii* leads to abnormal starch granule size and morphology

**DOI:** 10.1038/s41598-019-39506-6

**Published:** 2019-02-13

**Authors:** Justin Findinier, Sylvain Laurent, Thierry Duchêne, Xavier Roussel, Christine Lancelon-Pin, Stéphan Cuiné, Jean-Luc Putaux, Yonghua Li-Beisson, Christophe D’Hulst, Fabrice Wattebled, David Dauvillée

**Affiliations:** 1 University Lille, CNRS, UMR 8576–UGSF–Unité de Glycobiologie Structurale et Fonctionnelle, F-59000 Lille, France; 2 University Grenoble Alpes, CNRS, CERMAV, F-38000 Grenoble, France; 3 CEA, Institut de Biologie Environnementale et de Biotechnologie, Laboratoire de Bioénergétique et Biotechnologie des Bactéries et Microalgues, F-13108 Saint-Paul-lez-Durance, France

## Abstract

*Chlamydomonas reinhardtii* represents an ideal model microbial system to decipher starch metabolism. In this green algae, in cells growing in photosynthetic conditions, starch mainly accumulates as a sheath surrounding the pyrenoid while in cells subjected to a nutrient starvation, numerous starch granules are filling up the plastid stroma. The mechanisms underlying and regulating this switch from photosynthetic to storage starch metabolisms are not known. In this work, we have isolated a Chlamydomonas mutant strain containing a deletion in chromosome 2 which displays abnormal starch granule distribution. Under nitrogen starvation, this strain contains an additional starch granules population. These granules are twice as big as the wild-type granules and display characteristics of photosynthetic starch. Genetic and functional complementation analyses allowed us to identify the gene responsible for this original phenotype which was called *BSG1* for “Bimodal Starch Granule”. Possible roles of *BSG1* in starch metabolism modifications during the transition from photosynthetic to starved growth conditions are discussed.

## Introduction

Starch is the major storage polysaccharide in plants and accumulates in the form of insoluble granules inside the plastids of the plant cells. Starch is composed of two types of α-glucan polymers, amylose and amylopectin, which represent approximately 98–99% of the granule dry weight. The ratio of the two polymers varies according to the botanical origin of the starch but is generally comprised between 20–30% (dry weight) of amylose and 70–80% of amylopectin. Amylopectin consists of short linear chains of glucose units linked by α-1,4 glycosidic bonds and contains around 5% of branches at the α-1,6 position. Amylose is composed of long linear glucose chains containing less than 1% of branch points. The highly ordered organization of glucose chains in amylopectin confers specific physicochemical properties to this polysaccharide. Starch is semi-crystalline but the allomorphic type and degree of crystallinity vary depending of the botanical and/or genetic sources^1,2^. Several additional criteria distinguish starches depending on their botanical origins. They include the size of the granules (from 0.1 to up to 100 µm in diameter) or their shapes which can be ovoid, ellipsoidal, spherical, angular or lenticular^3–5^. Whilst our knowledge of the enzymatic reactions leading to starch synthesis has strongly improved during the last two decades, factors determining starch granules shape and size remain to be identified. Most starch accumulating organs contain one type of granule shape the size distribution of which is usually unimodal (i.e. particle size is more or less homogenously distributed around a unique major size value) with the notable exception of *Festucoideae* species such as wheat (*Triticum aestivum*), barley (*Hordeum vulgare*) or rye (*Secale cereale*). In these plants, starch granule size distribution is bimodal^6–8^; large disk-like granules (called A-type granules with an average diameter of about 25 µm in wheat) and smaller but spherical granules (called B-type granules with a diameter under 10 µm in wheat) accumulate in the endosperm^9^. Synthesis of the two types of starch granules is not simultaneous, as the B-granules appear several days after the large A-granule during grain filling^10,11^. While the majority of plants accumulates storage granules with specific size ranges and shape in sink tissues, the starch built in the source organs (called transitory starch) is unlikely to be identifiable to plant species as its shape is not specific and is likely to be determined by the space available at the site of synthesis^12^. In the unicellular green microalgae *Chlamydomonas reinhardtii*, different types of starch can be produced depending on growth conditions. Indeed, under unrestricted growth conditions, cells divide actively and accumulate photosynthetic (transitory) starch granules resembling the one found in higher plants source organs while a storage-like starch is synthesized under stress conditions, which harbors by many criteria the characteristics of endosperm storage starch^13–16^. While actively dividing, Chlamydomonas cells accumulate starch mostly around the pyrenoid, a specialized intraplastidial structure composed of aggregated ribulose-1,5-biphosphate carboxylase/oxygenase responsible for CO_2_ fixation. In such growth conditions, most starch is found organized as a sheath surrounding the pyrenoid^15,17^. In contrast, when subjected to a stress such as nitrogen starvation (-N), starch granules are massively accumulating in the stroma. After few days in these unfavorable conditions, starch granules are almost filling the cells^15^. This massive accumulation correlates with the loss of photosynthetic activity and disorganization of the thylakoid membranes^18^. The pyrenoid is hardly detected and no starch sheath can be detected around the remaining pyrenoid-like structure suggesting that it has been either degraded and/or modified^15^. Transitory and storage-like starches can also be distinguished by the structure of amylopectin (amylopectin chain length distribution (CLD) is different) and the amylose content (higher in storage-like starch)^15^. Even if both pyrenoidal and storage starches are synthesized by the same set of enzymes, differences in structure and composition reveals different enzymatic contributions in the processes. For instance, the study of a KO mutant of Chlamydomonas defective for one of the plastidial isoforms of starch-phosphorylase showed that this enzyme is required for correct storage starch synthesis but had no function in transitory starch synthesis^19^.

In this work, we have isolated and characterized a *Chlamydomonas reinhardtii* mutant carrying a small deletion on chromosome 2 induced by insertional mutagenesis. This mutant has a reduced rate of starch degradation and a modified amylopectin structure. When grown under nitrogen starvation, this mutant has a phenotype similar to that reported in cereals endosperm accumulating two populations of starch granules with distinct sizes. The first population contains starch granules similar to that of the wild-type while the second is composed of abnormally large granules with irregular shapes. Because of this original phenotype, the mutant strain was named *bsg1* for “bimodal starch granule distribution”. Genetic and functional complementation experiments allowed us to identify a candidate *BSG1* gene that was confirmed by the phenotypic characterization of a second mutant allele. Structural characterizations of both mutant granule populations and electron microscopy observations suggest different origins of the two types of starch granules. Possible function of the BSG1 protein leading to this unique phenotype of the mutant is discussed.

## Results

### Phenotypic characterization of the Chlamydomonas reinhardtii *bsg1-1* mutant

**Figure 1:**
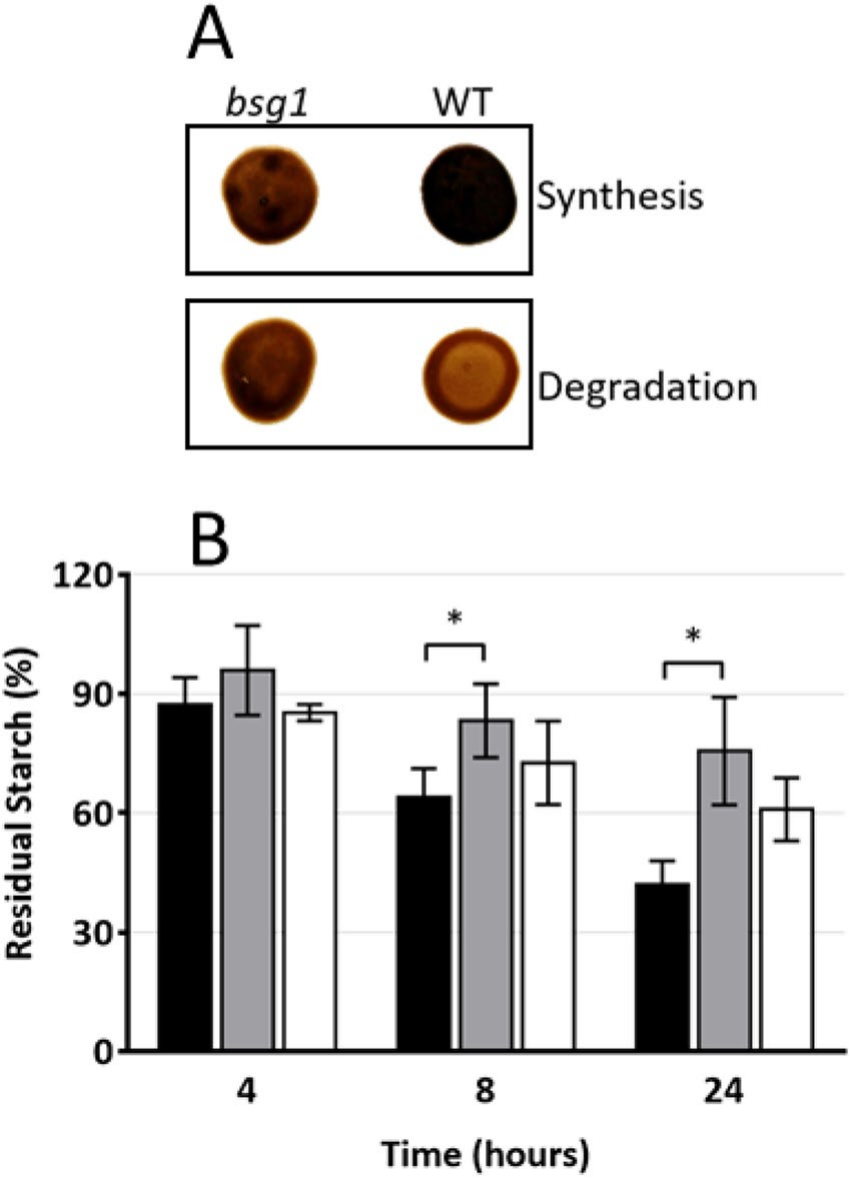
Phenotypic characterization of the *bsg1-1* mutant strain. (**A**) Two-step iodine screening. The WT and mutant strains were incubated on nitrogen starved medium for 5 days allowing massive starch accumulation and stained with iodine (upper panel, synthesis). Starvation was then alleviated and the cells incubated in the dark for 24 additional hours prior staining (lower panel, degradation). (**B**) Kinetic of starch mobilization in the dark. After a massive starch accumulation under nitrogen starvation for 5 days in the light, the cells were transferred into darkness after starvation removal and starch was assayed at each time point and expressed as a percentage of the initial amount for both the *bsg1-1* mutant (grey column), the isogenic wild-type strain (black column) and one complemented strain C1 (white column). Values correspond to means ± SD of three independent experiments *P < 0,05 by Student’s *t* test.

**Figure 2:**
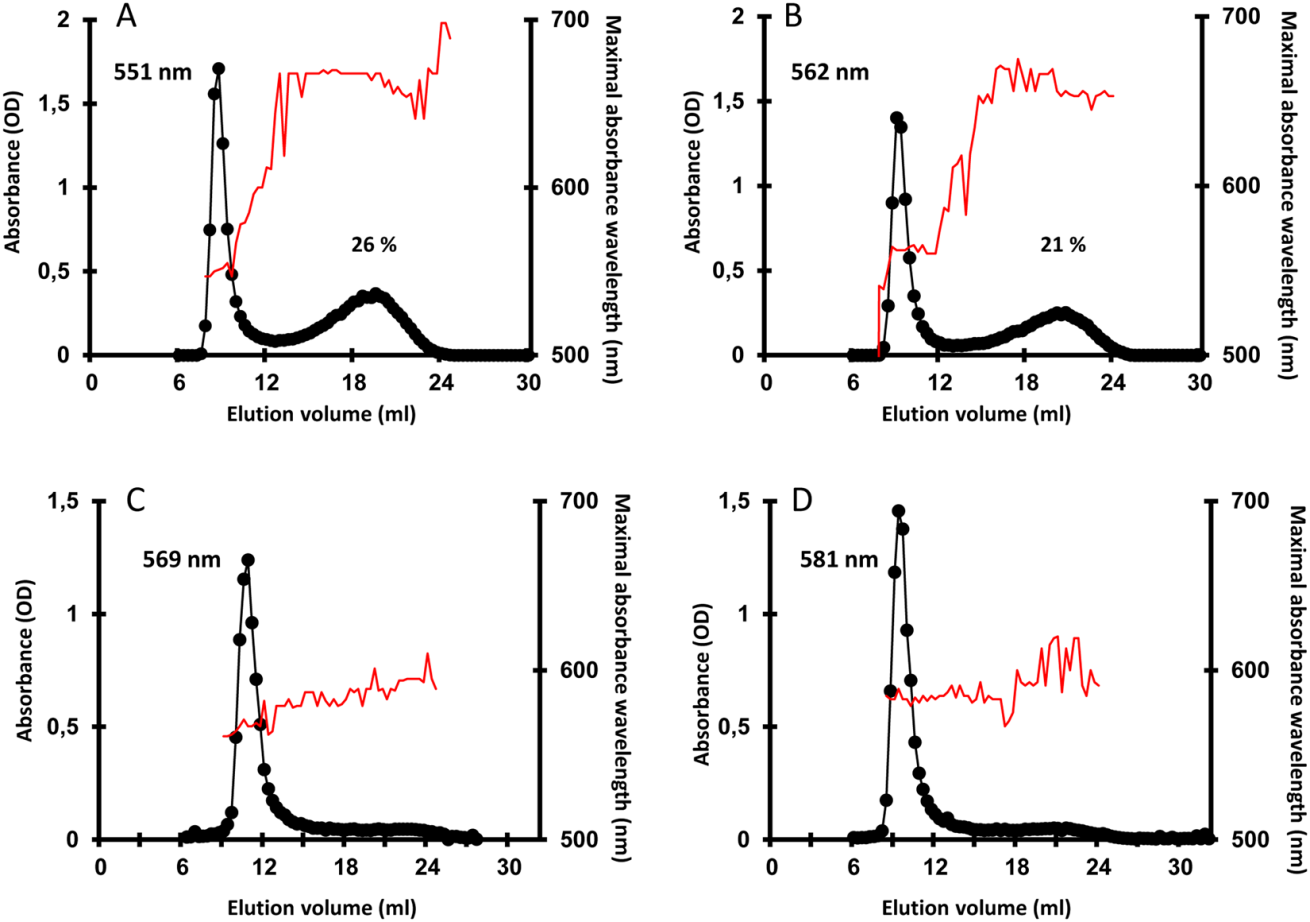
Separation of amylopectin and amylose on CL-2B chromatography. The optical density (black circles) was measured for each 300 µL fraction at λ_max_ (thin red line). All samples were loaded on the same column setup as described in^13^. Starches purified from nitrogen-starved culture (**A**,**B**) or exponentially growing cells (**C**,**D**) were analyzed. Plots (**A**,**C**) correspond to the chromatograms obtained with starch extracted from the wild-type strain 137C and (**B**,**D**) to the *bsg1-1* mutant. The amylopectin λ_max_ and the amylose percentage of each starch are indicated on the corresponding graphs.

We have recently constructed an insertional mutant library in *Chlamydomonas reinhardtii* wild-type strain 137C to identify new components of the starch degradation machinery^20^. From this mutant library, we have selected one mutant with an abnormal starch phenotype which was selected as a putative starch catabolism mutant^20^. Surprisingly, this mutant accumulated less starch than the wild type after a 5-day period in condition of massive starch accumulation. Indeed, this mutant showed a lighter color than the wild type when cell patches on Petri dishes were stained with iodine vapors (Fig. 1A). However, the staining intensity of this mutant remained the same even after 24 h of degradation while it was strongly reduced in the wild type (Fig. 1A). This phenotype was confirmed by determining the degradation rate (Fig. 1B) which was five times lower in the mutant compared to the wild type 137C (0.29 ± 0.14 µg starch degraded.10^−6^ cell.h^−1^ and 1.47 ± 0.10 µg starch.10^−6^ cell.h^−1^ respectively). Thus, after a 24-h period of degradation, the mutant still contains 76 ± 14% the initial starch amount (measured at the end of the massive accumulation period) while the wild type contains 42 ± 6% of starch. As suggested by iodine staining and surprisingly, even if starch degradation was impaired in the strain, it accumulated half as much starch as the wild type when grown under nitrogen starvation (Table 1). Under mixotrophic growth conditions, the mutant also accumulated less starch than the wild-type. In these growth conditions, the assessed difference was statistically significant (Table 1). Purified starches were analyzed by gel permeation chromatography on Sepharose CL-2B column. When starches were purified from nitrogen-starved cultures, both wild type and mutant strains accumulated amylose and amylopectin in similar ratio (amylose being about 20% of the total starch amount). However, upon iodine interaction, the λ_max_ of the amylopectin of the *bsg1-1* mutant was 10 nm higher than the wild type (Fig. 2A,B). A similar increase of the iodine-amylopectin λ_max_ of the mutant was observed when cells were grown in mixotrophic conditions (Fig. 2C,D). These modifications of the λ_max_ of the iodine-amylopectin complexes suggest that the structure of amylopectin is likely modified in the mutant.

**Table 1:** Starch deposition in the *bsg1* mutants.

Growth conditions	137C	*bsg1-1*	CC5325	*bsg1-2*
TAP	2.47 ± 0.27	1.72 ± 0.16^**^	3.24 ± 0.24	3.46 ± 0.25
TAP-N	60.70 ± 2.55	27.40 ± 2.65^***^	58.50 ± 4.20	61.60 ± 6.77

Starch amounts of both *bsg1-1* and *bsg1-2* mutant and their respective isogenic wild-type reference strains 137C and CC5325 were assayed in mixotrophic growth conditions (continuous light and acetate, TAP) or after five days under nitrogen starvation in continuous light (TAP-N). Values correspond to means ± SD of three biological replicates and are indicated in µg starch per million cells. ***P < 0,001 **P < 0.01 by Student’s *t* test.

**Figure 3:**
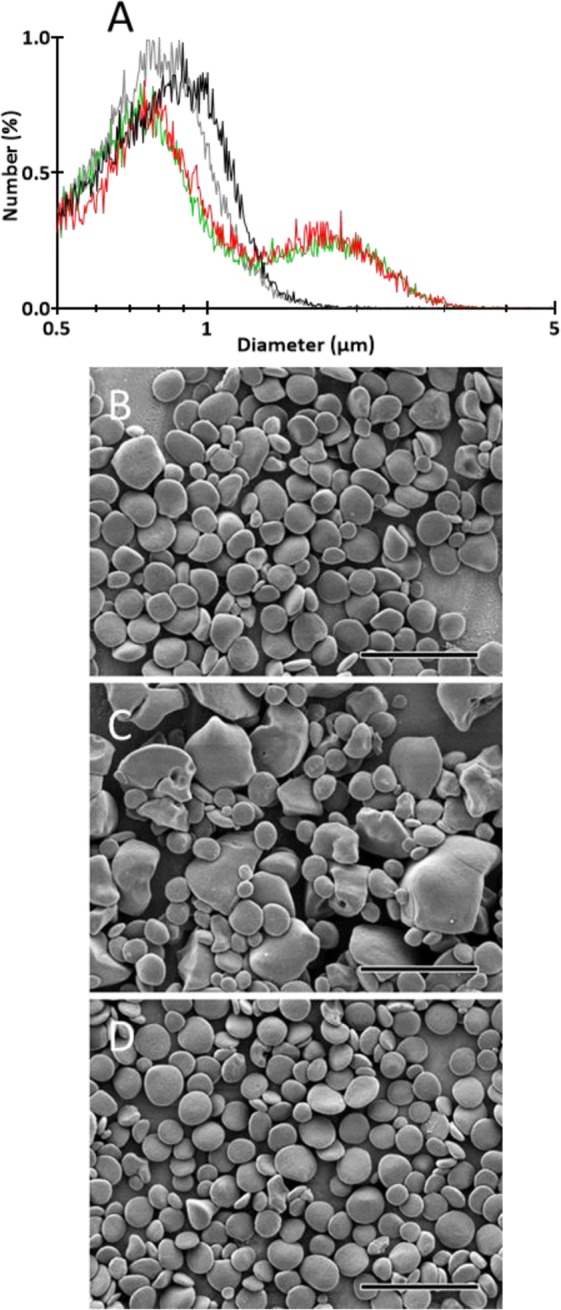
Starch granule size and morphology. Starch granules were extracted and purified from cells cultivated 5 days under nitrogen starvation and continuous light. (**A**) The starch granule size distribution was determined by analyzing 30,000 particles extracted for each genotype. The results are expressed in relative percentage (*y*-axis) of particles of a diameter ranging from 0.5 to 5 µm (*x*-axis, logarithmic scale). Starch from the wild-type 137C, the *bsg1-1* mutant, a complemented strain (C1) and a mutant strain transformed with an empty vector are represented in black, red, grey and green, respectively. (**B**–**D**) Starch granules were observed using SEM. Starch was extracted from the strains (**B**) 137C, (**C**) *bsg1-1* and (**D**) the complemented strain C1. Scale bar = 4 µm.

However, the most striking modification recorded in the mutant was the identification of two populations of starch granules with distinct average sizes that accumulate in the same cell cultivated in nitrogen starvation. In the wild type, starch granule size distribution shows a unimodal pattern with a maximum peak at 0.83 µm ± 0.04 in the wild-type (Fig. 3A). Two maximum peaks were detected in the mutant (Fig. 3A). The first peak was observed at a size comparable to that of the wild type (0.78 µm ± 0.04) while the second peak was at a size (1.84 µm ± 0.04) twice as large as the first peak. This original phenotype was confirmed by scanning electron microscopy (SEM) observations of native starch granules purified from nitrogen-starved cultures (Fig. 3B,C). Two populations (small and large) of starch granules could easily be seen in the mutant (Fig. 3C) while granule size was much more homogenous in the wild type (Fig. 3B).

### Identification of the mutation in *bsg1-1* and genetic analyses

**Figure 4:**
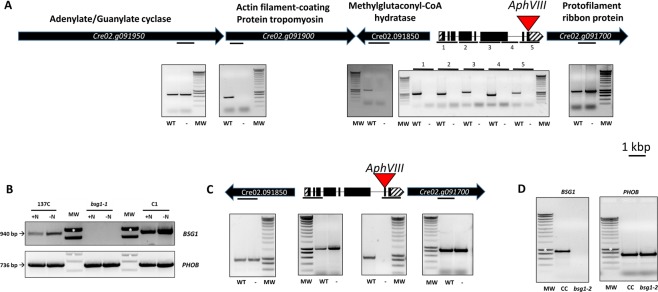
Characterization of the deletion in the *bsg1* mutant strains. (**A**) Schematic representation of the chromosome 2 in the *bsg1-1* mutant strain. The position of the insertion site of the paromomycin cassette into the *Cre02.g091750* locus is indicated in red (*AphVIII*). Specific primers for each locus surrounding *Cre02.g091750* were used to amplify a part of the corresponding gene (symbolized by a dark bar) from wild-type (WT) or mutant (−) genomic DNA. Five different primer pairs covering the whole *Cre02.g091750* gene were used to test the integrity of this locus. The description of each locus available on the phytozome website is indicated on the top of each gene. Scale bar = 1 kbp. (**B**) RT-PCR analysis. Total RNAs were extracted from cells in log-phase growing stage (+N) or after transfer in nitrogen free medium for 1 h (−N). One µg of total RNAs from the wild-type strain 137C, the original *bsg1-1* mutant and the complemented strain C1 were used to amplify a 940 bp fragment of the *Cre02.g091750* gene by RT-PCR. A 736 bp fragment of the starch phosphorylase gene (*PHOB*) was amplified as a control. (**C**) Schematic representation of the chromosome 2 in the *bsg1-2* mutant strain. The position of the insertion site of the paromomycin cassette into the *Cre02.g091750* locus is indicated in red (*AphVIII*). Specific primers for each locus surrounding *Cre02.g091750* were used to amplify a part of the corresponding gene (symbolized by a dark bar) from wild-type (WT) or mutant (*−*) genomic DNA. (**B**) RT-PCR analysis. One microgram of total RNA extracted from cells in log-phase growing stage from the wild-type strain CC5325 (CC) and the *bsg1-2* mutant were used to amplify a 940 bp fragment of the *Cre02.g091750* gene by RT-PCR. A 736 bp fragment of the starch phosphorylase gene (*PHOB*) was amplified as a control. All the primers used are described in Supplementary Table S2. MW: Eurogentec Smartladder, the white stars correspond to the 1 kbp band of the marker.

The *bsg1-1* mutant was produced by insertional mutagenesis using a paromomycin resistance cassette introduced in the wild-type strain 137C^20^. Thermal Asymmetric InterLaced (TAIL) PCR on *bsg1-1* genomic DNA allowed the localization of one insertion of the cassette into chromosome 2. We were able to amplify by PCR a fragment containing 87 bp corresponding to the end of the paromomycin cassette and 446 bp of the flanking genomic sequence when the genomic DNA purified from the mutant was used. This flanking sequence located at the 3′ end of the inserted resistance gene, matches to the 11 last coding nucleotides of the *Cre02.g091750* locus and the following genomic region (Phytozome V12.1; https://phytozome.jgi.doe.gov/). It is well known that insertional mutagenesis can lead to multiple insertions of the resistance cassette in the genome and to chromosomic rearrangements such as deletions^21–23^. Using specific primers annealing either to *Cre02.g091750* or to the adjacent loci, the *bsg1-1* mutant was shown to carry a deletion of approximatively 15 kbp at the insertion site (Fig. 4A). In the mutant, the *Cre02.g091900* (described as an actin filament-coating protein tropomyosin), *Cre02.g091850* (annotated as a methylglutaconyl-CoA hydratase) and *Cre02.g091750* (described as a phosphoglucan, water dikinase, PWD) genes were deleted. Glucan, water dikinases (GWD) and phosphoglucan, water dikinases (PWD) are key enzymes of the initial steps of starch degradation^24,25^. These starch phosphorylating enzymes are required for efficient starch degradation in leaves and share a common catalytic mechanism in which a conserved histidine residue is involved in the transfer of the β-phosphate group of ATP to the glucan^24,26–28^. The homology of the protein encoded by the *Cre02.g091750* locus with dikinases only implied a restricted part of the polypeptide (from amino acids 72 to 150) corresponding to a carbohydrate-binding domain (CBM20) at the N-terminal extremity of the protein and sharing 29% identity (65% similarity) with the CBM module found in the Arabidopsis PWD (*At5g26570*). Moreover, this homology didn’t cover the part of PWD containing the essential catalytic histidine residue. When the complete protein sequence was used to try to find homologs in higher plants, all attempts failed. The only homologs were identified in microalgae close relatives to Chlamydomonas such as Volvox although homology with the Chlamydomonas protein was low (the Volvox protein *Vocar.0001s1160.1*, which shares the highest homology degree displays only 28% similarities with the Chlamydomonas protein). The lack of expression of *Cre02.g091750* in the *bsg1-1* mutant was confirmed by RT-PCR performed on total RNAs samples prepared from either log-phase growing-cells or cells submitted to nitrogen starvation for 1 hour (Fig. 4B).

We then engaged in genetic analysis to check the link between the pleiotropic phenotype of the *bsg1-1* mutant and the deletion in chromosome 2. In each tetrad obtained from a cross with the wild-type reference strain 37 (see methods), two segregants were resistant to paromomycin and two sensitive indicating that only one functional copy of the mutational paromomycin cassette has been inserted in the genome of the mutant. The consequence of the insertion of the paromomycin cassette at the *Cre02.g091750* locus was checked in all seven tetrads. A mendelian segregation was obtained with two spores carrying the wild type allele and two carrying the chromosomal deletion (Supplementary Fig. S1A). Each progeny resistant to paromomycin carried the deletion in chromosome 2 while all sensitive progeny contained a wild-type copy of *Cre02.g091750*.

Starch from nitrogen-starved cultures of each meiotic segregant was then extracted and subjected to the same complete set of analysis that was performed on the original mutant.

The cosegregation of the starch phenotype and the mutation at the *Cre02.g091750* locus was then investigated by the analysis of tetrads progeny. First, we have determined that, such as the original *bsg1-1* mutant, all mutant progeny produced amylopectin with altered structure. Indeed, iodine-amylopectin λ_max_ is increased by about 10 nm (563 nm ± 7 and 554 nm ± 3 in mutant and wild type progeny respectively; statistically significant at p < 0.001 with Student’s *t*-test). Second, the starch degradation phenotype was scored in the progeny. The segregation pattern fitted that expected for a single mendelian trait (Supplementary Fig. S1B). Once again, the defect of starch degradation was only recorded in the progeny carrying the deletion in chromosome 2. Indeed, all mutant segregants still contained 71 ± 15% of their initial starch content after 24 h in darkness, while all wild-type segregants contained only 34 ± 9% (Supplementary Fig. S1C). Finally, the presence of the two populations of small and large starch granules was systematically observed in the mutant progeny while only one population of granules of regular size distribution was observed in the wild-type (Fig. S1D).

However, while the *bsg1-1* mutant accumulated half as much starch as the wild type 137C, we were unable to discriminate between the mutant and wild-type progeny when the amount of starch accumulated under nitrogen starvation was considered. A large difference in the amounts of starch accumulated in the strains was observed leading to an average of 50 ± 30 µg starch.10^−6^ cell in the mutants and 41 ± 19 µg starch.10^−6^ cell in the wild type, for cells cultivated in the same conditions.

### Functional complementation of the *bsg1* mutant

One of the 3 genes lacking in *bsg1-1* encodes a protein containing a carbohydrate binding module with a putative 50 amino acids transit peptide (as determined by ChloroP; http://www.cbs.dtu.dk/services/ChloroP/) annotated as PWD. We suspected that the starch phenotype of the *bsg1-1* mutant was the consequence of the specific lack of this gene. We have tested this hypothesis by reintroducing a wild-type copy of this gene in the mutant. To this end, the 3.7 kbp region between the start and the stop codon of the gene *Cre02.g091750* was amplified and cloned under the strong *PSAD* (a subunit of photosystem I) promoter into the pSLHyg plasmid containing the *Aph7*″ gene conferring hygromycin resistance^29^. The transformants were selected on TAP plates containing both paromomycin and hygromycin. Starch of twenty transformed strains resistant for both paromomycin and hygromycin was analyzed for particle size distribution. Eighteen of them displayed a unimodal starch granule distribution with peak size of 0.75 ± 0.03 µm (Fig. 3A). This result was confirmed by SEM observations of purified starch granules (Fig. 3D). Note that the restoration of the wild-type phenotype in the complementing strain C1 was correlated to the expression of *BSG1* mRNA as revealed by RT-PCR analysis (Fig. 4B). However, complementation of the starch phenotype was not complete in these eighteen transformed strains. Indeed, the increase of the λ_max_ of the iodine-amylopectin recorded in the mutant was still observed in four independent complemented strains tested (561 nm ± 1). Furthermore, the complemented strains still degraded starch with a rate similar to that of the original mutant and not the wild type strain (0.51 ± 0.21 µg starch degraded.10^−6^ cell.h^−1^; Fig. 1B). Moreover, the lower starch content measured in the original mutant was still detected in the complemented strains tested. These strains contained an average of 22 ± 8 µg starch.10^−6^ cell when grown under nitrogen starvation.

In parallel, the original *bsg1* mutant was also transformed with an empty linearized pSLHyg vector. Over 13 transformants resistant to both paromomycin and hygromycin, none recovered the wild-type phenotype even partially, and still displayed a bimodal starch granule distribution (Fig. 3A).

### Phenotypic characterization of a second mutant strain at Cre02.g091750

While a normal starch granule distribution was observed in the complemented strains expressing a wild-type copy of the *Cre02.g091750* gene, several phenotypes identified in the original *bsg1-1* mutant was still detected in these strains (slow degradation rate, amylopectin high λ_max_). To resolve these discrepancies, we characterized the phenotype of a second insertional mutant at the same locus. The LMJ.RY0402.102699 strain from the CLiP library contains a copy of the paromomycin cassette inserted into its penultimate exon (Fig. 4C). At the contrary of the *bsg1-1* mutant, no deletion of the surrounding loci could be evidenced in this mutant strain (Fig. 4C). This strain is not expressing the *Cre02.g091750* locus as evidenced by RT-PCR (Fig. 4D). Thus, this strain defines a second mutant allele (called *bsg1-2*) at the *Cre02.g091750* locus. We performed a complete phenotypic characterization of this strain and its wild-type counterpart (CC5325) to discriminate between the phenotypes due to a defect in *BSG1* and additional phenotypes that could be the consequence of the deletion of the neighboring genes evidenced in the original *bsg1-1* mutant strain.

The *bsg1-2* mutant accumulated similar starch amounts than the wild type CC5325 in both growth conditions tested (mixotrophy or nitrogen starvation, Table 1). The degradation rates measured during starch degradation kinetics did not reveal a defect for the polysaccharide mobilization in this mutant (1.62 ± 0.34 µg starch degraded.10^−6^ cell.h^−1^ and 1.77 ± 0.39 µg starch.10^−6^ cell.h^−1^ for the *bsg1-2* mutant and the wild-type reference CC5325 respectively).

**Figure 5:**
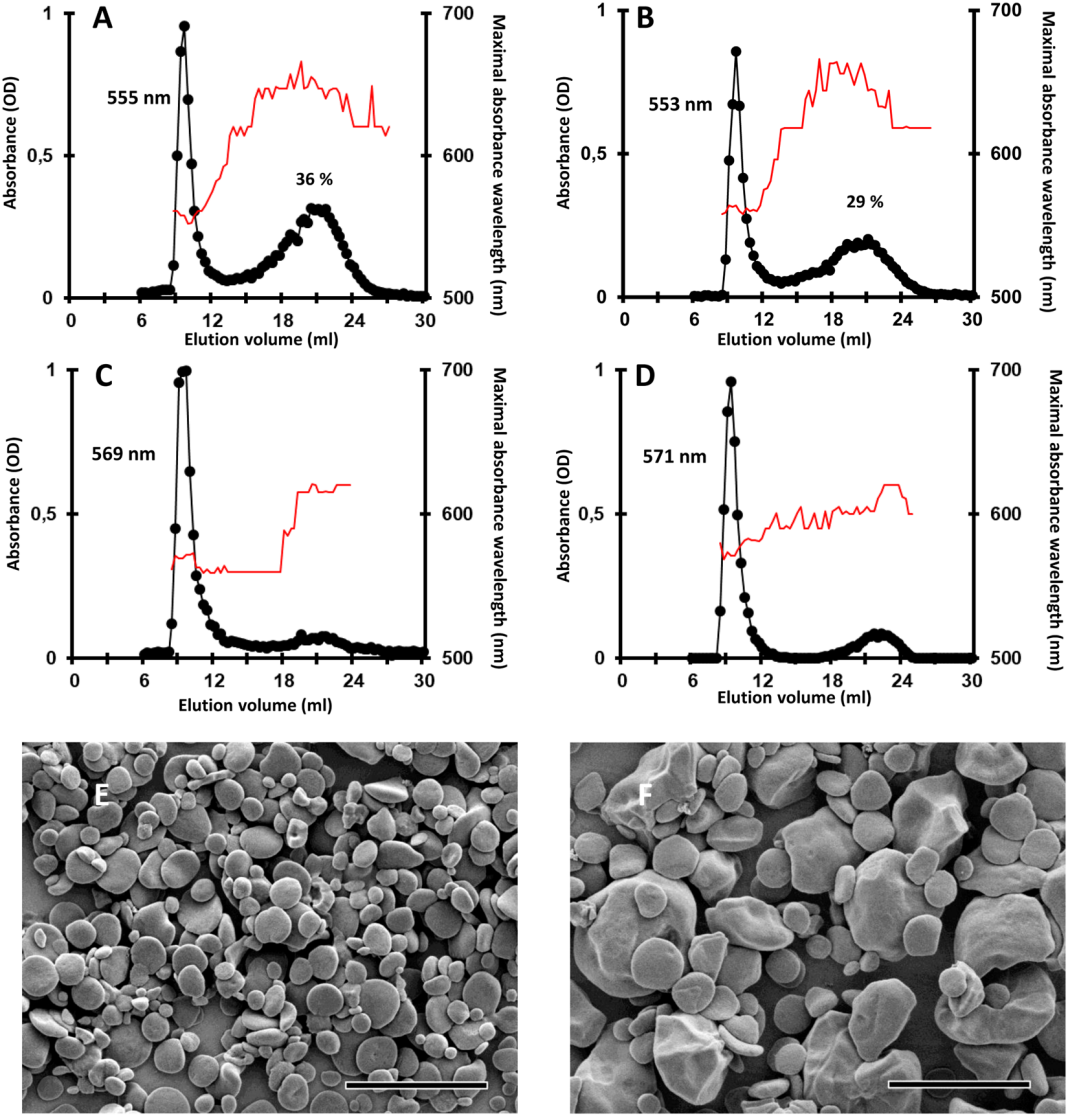
Phenotypic characterization of starches produced by the *bsg1-2* mutant strain. Starches purified from the *bsg1-2* mutant strain and its reference wild-type strain CC5325 were analyzed on CL-2B gel permeation chromatography The optical density (black circles) was measured for each 300 µL fraction at λ_max_ (thin red line). All samples were loaded on the same column setup as described in^13^. Starches purified from nitrogen-starved culture (**A**,**B**) or exponentially growing cells (**C**,**D**) were analyzed. Plots (**A**,**C**) correspond to the chromatograms obtained with starch extracted from the wild-type strain CC5325 and (**B**,**D**) to the *bsg1-2* mutant. The amylopectin λ_max_ and the amylose percentage of each starch are indicated on the corresponding graphs. (**E**,**F**) Starch granules from nitrogen starved cultures of the wild-type CC5325 (**E**) and the *bsg1-2* mutant (**F**) were observed using SEM. Scale bar = 4 µm.

Starches purified from both strains and from both growth conditions were analyzed by gel permeation chromatography on Sepharose CL-2B column (Fig. 5A–D). No significant difference could be evidenced between the starches produced by the two strains as evidenced by the similar amylopectin λ_max_ values recorded for these two strains in both conditions (Fig. 5). Nonetheless, the *bsg1-2* mutant strain was accumulating two starch granule populations under nitrogen starvation (Fig. 5F) while only the small population could be detected in its wild-type counterpart CC5325 (Fig. 5E).

### Characterization of the two starch granules populations accumulated in the *bsg1* mutants

**Figure 6:**
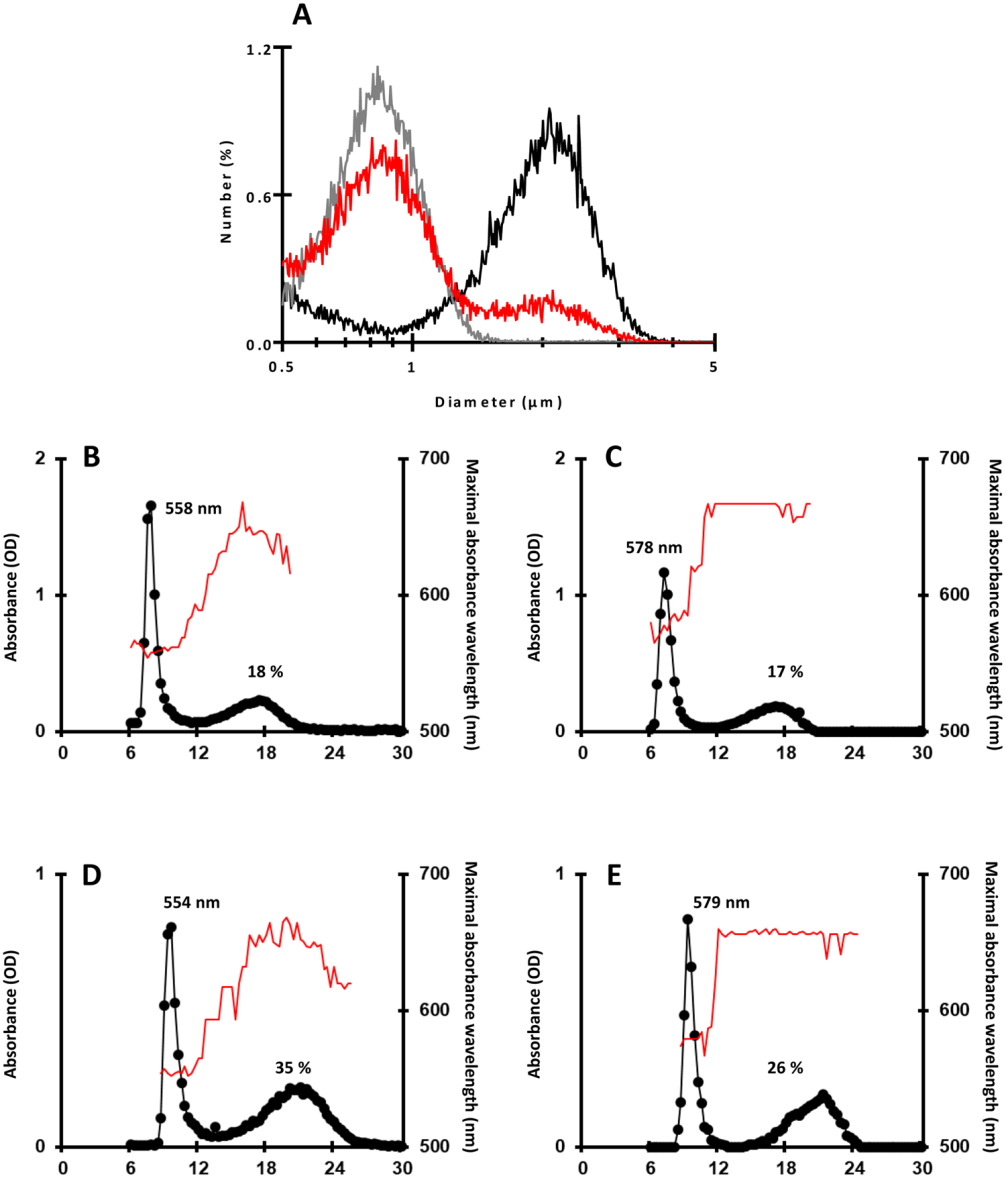
Separation and characterization of the two starch granules populations in *bsg1*. (**A**) The two starch granules populations found in *bsg1-1* were separated through repetitive filtering and the semi-purified fractions were analyzed on the coulter counter. The starch granule distributions of the starch produced by *bsg1-1* (red), the fraction corresponding to the small granules (grey) and the fraction enriched in large granules (black) are shown. (**B**,**C**) Separation of amylopectin and amylose on CL-2B chromatography. The optical density (black circles) was measured for each 300 µl fraction at λmax (unbroken red thin line). The chromatograms obtained with the small (**B**) and the large (**C**) starch granules are displayed. The amylopectin λ_max_ and the amylose percentage of each polysaccharide are indicated on the corresponding graphs. The same procedure was used on starch purified from the *bsg1-2* mutant strain grown under nitrogen starvation. The chromatograms corresponding to the small and the large starch granules produced by this mutant strain are displayed in (**D**,**E**) respectively.

In *Festucoideae* species such as wheat, the endosperm starch is made of large A and small B granules that differ in composition, CLD of amylopectin, and relative crystallinity^30–33^. In order to verify whether such differences could arise between the two populations of granules in the *bsg1* mutants, the small and large granules were separated (Fig. 6A,B; see methods). Then, each fraction was subjected to gel permeation chromatography (Sepharose CL-2B). The small starch granules from *bsg1-1* were similar to that of the mutant starch when cultivated in nitrogen-starvation condition with a λ_max_ of the iodine-amylopectin complex of 558 nm (Figs 2B and 6B). Conversely, the λ_max_ of the iodine-amylopectin complex of the large granules (578 nm) was similar to that of the *bsg1-1* mutant starch obtained from cells cultivated in mixotrophic conditions (Figs 2D and 6C). Interestingly, this starch contains a significant amount of amylose (17 ± 1%; n = 3) albeit it is usually low in mixotrophic starch (corresponding to starch granules found around the pyrenoid in such cell growth conditions). A similar pattern was observed for both small and large granules purified from the *bsg1-2* mutant. The small granules (Fig. 6D) displayed the same chromatographic profile and amylopectin λ_max_ than the corresponding mutant starch produced under nitrogen starvation (Fig. 5B) while the large granules (Fig. 6E) were harboring a high amylopectin λ_max_ value (579 nm) close to the value recorded for starch produced by the mutant in mixotrophic conditions (Fig. 5D). As it was recorded for the *bsg1-1* mutant, the large granules produced by *bsg1-2* contain a significant amount of amylose (Fig. 6E).

**Figure 7:**
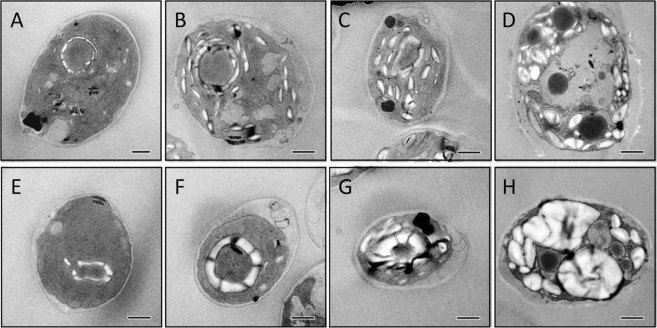
Transmission electron microscopy of thin sections of Chlamydomonas cells. The wild-type strain 137C (**A**–**D**) and the *bsg1-1* mutant (**E**–**H**) were visualized before (**A**,**E**) or after 8 h (**B**,**F**), 24 h (**C**,**G**) or 5 days (**D**,**H**) of nitrogen starvation. Scale bars = 1 µm.

Since the large starch granules in both *bsg1* mutants were only detected after the cells were submitted to nitrogen starvation, we have analyzed the morphology of the starch granules during the switch between mixotrophic to nitrogen-starvation conditions. To this end, cells from the *bsg1-1* mutant were first grown for 3 days under continuous light in TAP medium and then transferred to nitrogen free medium (TAP-N). Cells were visualized 8, 24 and 120 h after the switch. When starved of nitrogen source, Chlamydomonas cells engage in a scavenging mechanism that yields to the formation of non-photosynthetic cells with disorganized thylakoid membranes and in which the pyrenoid is slowly degraded. In the wild type, the pyrenoidal starch sheath was unchanged after 8 h of starvation (Fig. 7A,B) but became barely detectable after 24 h (Fig. 7C). In the meantime, numerous new starch granules were already detected in the stroma after 8 h under starvation (Fig. 7B). Conversely, after 8 h of starvation, the starch sheath was enlarged (Fig. 7E,F) in the *bsg1-1* mutant and was still clearly detected even after 24 h of starvation (Fig. 7G). Few new starch granules were also detected in the stroma of the mutant strain 8 h after starvation (Fig. 7F). After 24 h of starvation, the two starch granule populations were clearly visualized in the mutant and the enlarged pyrenoidal starch appeared bigger than the stromal starch granules (Fig. 7G). After 5 days of starvation, the differences between the mutant and the wild type were clearly evidenced by the presence of abnormally large starch granules detected only in the mutant cells (Fig. 7D,H). The TEM images of 20 independent cells cultivated for 5 days under nitrogen starvation were also used to determine the average number of starch granules per cell section. Interestingly, the number of starch granules per cell section was significantly decreased in the *bsg1-1* mutant compared to the wild type (11 ± 4 starch granules in the mutant and 30 ± 8 starch granules in the wild type).

## Discussion

### The Chlamydomonas reinhardtii *bsg1* mutants accumulate abnormally large starch granules

In this work, we have isolated a *Chlamydomonas reinhardtii* mutant strain, produced by insertional mutagenesis with a paromomycin resistance cassette. This mutant was selected for reduced starch degradation (Fig. 1). The strain contains a 15 kb deletion in chromosome 2 leading to the deletion of at least 3 different loci (Fig. 4). It also accumulates a low amount of starch with a modified structure (increased λ_max_ of the iodine-amylopectin complex). However, the most striking phenotype of this mutant stands on the accumulation of two starch granule populations of different sizes when the cells were grown under nitrogen starvation. Cosegregation analysis performed on tetrads arising from a cross between the original *bsg1-1* mutant and a wild type reference strain revealed a segregation as a single mendelian trait and cosegregation between this deletion and the defect in starch degradation, the modified amylopectin structure and the bimodal starch granules distribution but not to the low starch content phenotype. As no statistical difference could be observed between the amount of starch between the wild type and the mutant strains in the meiotic progeny of the cross, this phenotype cannot be linked to the mutation but appears to be dependent of the genetic background of the strains. This was confirmed by the *bsg1-2* mutant strain that accumulates similar amounts of starch than the isogenic wild-type reference strain CC5325.

Functional complementation experiments allowed us identifying the gene responsible for the unusual starch granules sizes distribution. The *Cre02.g091750* locus encodes a predicted protein of 792 amino acids for which no orthologous gene can be identified in higher plant genomes. The few proteins sharing low homology with the Chlamydomonas polypeptide was only found in phylogenetically close microalgae such as Volvox. The restricted distribution of this protein family may suggest an involvement in a metabolic pathway specific to microalgae. Concerning the defect in starch mobilization and the abnormal starch structure (high amylopectin λ_max_) recorded in the original *bsg1-1* mutant, we did not observe the restoration of a wild-type phenotype in the complemented strains. Moreover, these phenotypes were not recorded in the *bsg1-2* mutant strain in which only the *Cre02.g091750* gene is deleted. It seems likely these phenotypes are the consequence of the deletion of one of the adjacent genes of the *Cre02.g091750* locus. A good candidate could be *Cre02.g091850* (annotated as a methylglutaconyl-CoA hydratase and putatively involved in the leucine degradation pathway) as this kind of mutation may affect the whole cellular metabolism and consequently affect starch metabolism. The responsibility of this gene could be tested by introducing back a wild-type copy in the *bsg1-1* mutant strain.

### *BSG1* may play an important function required for pyrenoidal to storage starch metabolism transition

While the precise function of *BSG1* in starch metabolism is not fully uncovered, its importance in the process leading to the switch from pyrenoidal to storage starch metabolism is clearly demonstrated in this work. This protein contains a putative transit peptide of 50 amino acids as determined by ChloroP and a carbohydrate binding module extending from amino-acids 72 to 150 (CBM20). We have also found (by the use of three independent methods^34–37^) consistent evidences for the presence of a coiled-coil domain downstream the CBM20 into the BSG1 protein sequence. The strength of the predictions were in all cases strong and involved the same region extending from residue 302 to 380 (Supplementary Table S1). Recently, a new class of proteins containing coiled-coil domain and carbohydrate binding module (CBM48) were shown to play a role for efficient starch synthesis in Arabidopsis. This is the case of PTST (Protein Targeting to STarch), which was shown to be involved in the correct targeting of granule bound starch synthase (GBSS, responsible for the synthesis of amylose) to the starch granule^38^. Two PTST homologs (PTST2 and PTST3) were identified in Arabidopsis thanks to their sequence similarity with PTST^39^. The phenotype of the corresponding single and double mutants suggested an important role of both proteins in starch metabolism^39^. PTST2 and 3 were proposed as interacting with soluble starch synthase 4 (SS4) which is a major factor controlling the initiation of starch granule synthesis^40–42^. In rice, FLO6 (a PTST2 ortholog) was proposed to interact with the debranching enzyme ISA1 and to influence granule morphology and grain starch content^43^. More recently, proteins containing coiled-coil domain typically involved in protein/protein interactions^44^ but without CBM were shown as mediating interactions between proteins required for correct starch synthesis. This is the case for PII1/MRC and MFP1 that were shown as essential for normal starch granule initiation in Arabidopsis^45,46^. Furthermore, with regard to sequence/structure relationships and carbohydrate binding, the CBM48 family is closely related to the CBM20 family that is found in the Chlamydomonas protein^47^. Finally, BSG1 was recently shown to be one of the 190 proteins present in the Chlamydomonas pyrenoid proteome^48^ suggesting a role in pyrenoidal starch metabolism. The large starch granules found in the *bsg1* mutants grown under nitrogen starvation have a high λ_max_ of the iodine-amylopectin complex that is characteristic of pyrenoidal starches (Fig. 6C,E). Moreover, these abnormally large granules seem to stem from the pre-existing pyrenoidal starch sheath as evidenced by the micrographs of the cells transferred from mixotrophic to nitrogen-starved growth conditions (Fig. 7). Because of the presence of a coiled-coil domain and a CBM20 domain in BSG1, a similar role to the one described for the PTST or PII1/MRC/MFP proteins may be suggested. When Chlamydomonas cells are subjected to unfavorable growth conditions, starch metabolism rapidly switches from restricted pyrenoidal to massive storage synthesis. During this transition, BSG1 may be involved in the correct targeting of degradation enzymes required for pyrenoidal starch degradation and its replacement by larger stromal starch granules. This defect in pyrenoidal starch degradation would explain the enlargement of the granules observed in the *bsg1* mutants. Another possible scenario would be that BSG1 is required for initiating new starch granules in the stroma when the carbon flux is massively oriented toward the massive accumulation of storage molecules. When BSG1 is lacking, the number of new initiation events decreases and the pyrenoidal starch is then used as a primer for massive starch synthesis. The decreased number of starch granules detected in the mutant is consistent with both hypotheses. The precise function of this protein in this important physiological adaptation of the cells to unfavorable conditions will require further investigations. It will be interesting to determine the putative interacting partners of BSG1 to unravel its precise role in the regulation of starch metabolism in various growth conditions.

## Methods

### Chlamydomonas strains, growth, crosses and media

The two wild-type strains of *Chlamydomonas reinhardtii* used in this study were 37 (*mt* + *pab2 ac14*) and 137C (*mt− nit1 nit2*). Standard media^47^ and growth conditions have been previously described^13,49^. Briefly, all experiments unless specified were carried out in continuous light (40 µE m^−2^ s^−1^) in the presence of acetate at 24 °C in liquid cultures that were shaken without air or CO_2_ bubbling. Late-log phase cultures were inoculated at 10^5^ cells mL^−1^ and harvested at 2–3.10^6^ cells mL^−1^. Nitrogen-starved cultures were inoculated at 5.10^5^ cells mL^−1^ and were harvested after 5 days at a final density of 1 to 2.10^6^ cells mL^−1^. For starch degradation kinetics experiments, the cells harvested after 5 days under nitrogen starvation were collected in sterile conditions and concentrated ten times by resuspension in TMP (TAP medium without acetate) liquid medium before incubation in the dark for 4, 8 and 24 h. Starch was purified from these samples and the amount of remaining polysaccharide was assayed and compared to the initial starch amount assayed before the switch to darkness. The 2 steps iodine screening procedure for identification of starch degradation mutants on solid medium has been described elsewhere^20^. The *bsg1-1* mutant was isolated after nuclear genome transformation of the wild-type strain 137C using a paromomycin cassette as described in^20^. The *bsg1-2* mutant (LMJ.RY0402.102699) together with its background strain CC5325 were purchased from the CLiP collection at https://www.chlamylibrary.org ^50^. For tetrads analyses, the *bsg1-1* mutant was crossed with the wild-type strain 37 (strain 37 is a *Chlamydomonas reinhardtii* segregant with a genetic background derived from the wild-type reference) and the meiotic progeny was analyzed in 7 complete tetrads. Auxotrophy and mating type of each progeny were determined to ensure that segregation occurred correctly in each tetrad.

### Determination of starch levels, starch purification, amylopectin structures

A full account of amyloglucosidase assays, starch purification and λmax (maximum wavelength of the iodine polysaccharide complex) measurements can be found in^13^. Separation of starch polysaccharides by gel permeation chromatography on a Sepharose CL-2B column was performed as in^51^. Student’s t test were carried out using the online tool available at http://www.physics.csbsju.edu/stats/t-test.html.

### Molecular techniques

All oligonucleotides used in this study are described and listed in Supplementary Table S2.

Total DNA from Chlamydomonas was purified as previously described^52^. Thermal asymmetric interlaced (TAIL) PCR was used to obtain the DNA sequence flanking the paromomycin cassette insertion site using the primers and cycling parameters previously described^20^. Homology searches were performed using the Phytozome website^53^. Total RNA from Chlamydomonas was extracted following an established protocol^54^. An aliquot of 1 μg of total RNA was used for reverse transcription (RT)-PCR analysis using the One Step RT PCR-Kit (Qiagen) following the manufacturer’s instructions. The complete Chlamydomonas *Cre02.g091750* locus was amplified as a 3.7 kb fragment using the Dynazyme EXT DNA polymerase (Thermo Fisher scientific) and was subsequently cloned using the TOPO TA Cloning Kit (Invitrogen). This genomic DNA fragment was transferred into pSLHyg^19^ in the *Eco*RV site downstream of the *PSAD* promoter and 5′-UTR sequence. For functional complementation, the mutant strain was transformed either with an empty pSLHyg vector conferring resistance to hygromycin (20 μg ml^−1^) or with the same vector containing the Chlamydomonas gene, using the glass bead transformation method^55^. The identification of the deletion in chromosome 2 in the meiotic progeny was performed through PCR amplification of a 1078 bp fragment of the *Cre02.g091750* locus using the BSGF and BSGR primers (Supplementary Table S2). Fragments of the neighboring genes were amplified from wild-type and mutant DNAs at optimal temperature with specific primers described in Supplementary Table S2. Dynazyme EXT DNA polymerase was used for each amplification following the manufacturer’s recommendation at optimal temperature (see Supplementary Table S2) in the presence of 5% DMSO.

### Starch granule size distribution, Transmission electron microscopy (TEM) and Scanning electron microscopy (SEM)

Small and large starch granule populations produced by both *bsg1* mutant strains were separated as follows. Purified starches (10 mg) were purified from nitrogen starved cultures of each strain and filtered through a 0.8 µm pore size filter. The flow-through containing only small granules and the retaining fraction highly enriched in large granules were checked in a multisizer 4 Coulter-counter (Beckman). Purified starch granules (approximatively 1 µg) from nitrogen starved cultures in 10 mL of IsoFlow Sheath (Beckman Coulter) were analyzed in a multisizer 4 Coulter-counter (Beckman) with a 20 µm aperture tube. The Multisizer software was set to determine the size of 30,000 particles ranging from 0.5 to 5 µm. 300 bins are logarithmically spaced between 0.5 and 5 µm (X-axis) and the size frequency distribution was expressed as relative percentage of total amount (Y-axis).

The cells were fixed in suspension with glutaraldehyde, post-fixed with osmium tetroxide and embedded in Epon resin. 70 nm-thin sections were cut with a diamond knife in a Leica UC6 ultramicrotome and post-stained with uranyl acetate and lead citrate. The sections were collected on carbon-coated copper grids and observed with an FEI-Philips CM200 transmission electron microscope operating at 200 kV. Digital images were recorded with a TVIPS TemCam F216 camera.

Droplets of purified starch granule suspensions were allowed to dry on freshly cleaved mica. The specimens were coated with Au/Pd and secondary electron images were recorded with an FEI Quanta 250 scanning electron microscope equipped with a field emission gun and operating at 2 kV.

## Supplementary information

Supplementary information
