# Supplementary material for: Deletion of BSG1 in Chlamydomonas reinhardtii leads to abnormal starch granule size and morphology
Source: Sci Rep. 2019 Feb 13;9:1990. doi: 10.1038/s41598-019-39506-6 (PMC6374437; doi:10.1038/s41598-019-39506-6)

Supplementary information

Deletion of *BSG1* in *Chlamydomonas reinhardtii* leads to abnormal starch granule size and morphology.

Justin Findinier[1,#], Sylvain Laurent[1,#], Thierry Duchêne[1], Xavier Roussel[1], Christine Lancelon-Pin[2], Stéphan Cuiné[3], Jean-Luc Putaux[2], Yonghua Li-Beisson[3], Christophe D'Hulst[1], Fabrice Wattebled[1] and David Dauvillée[1,*]

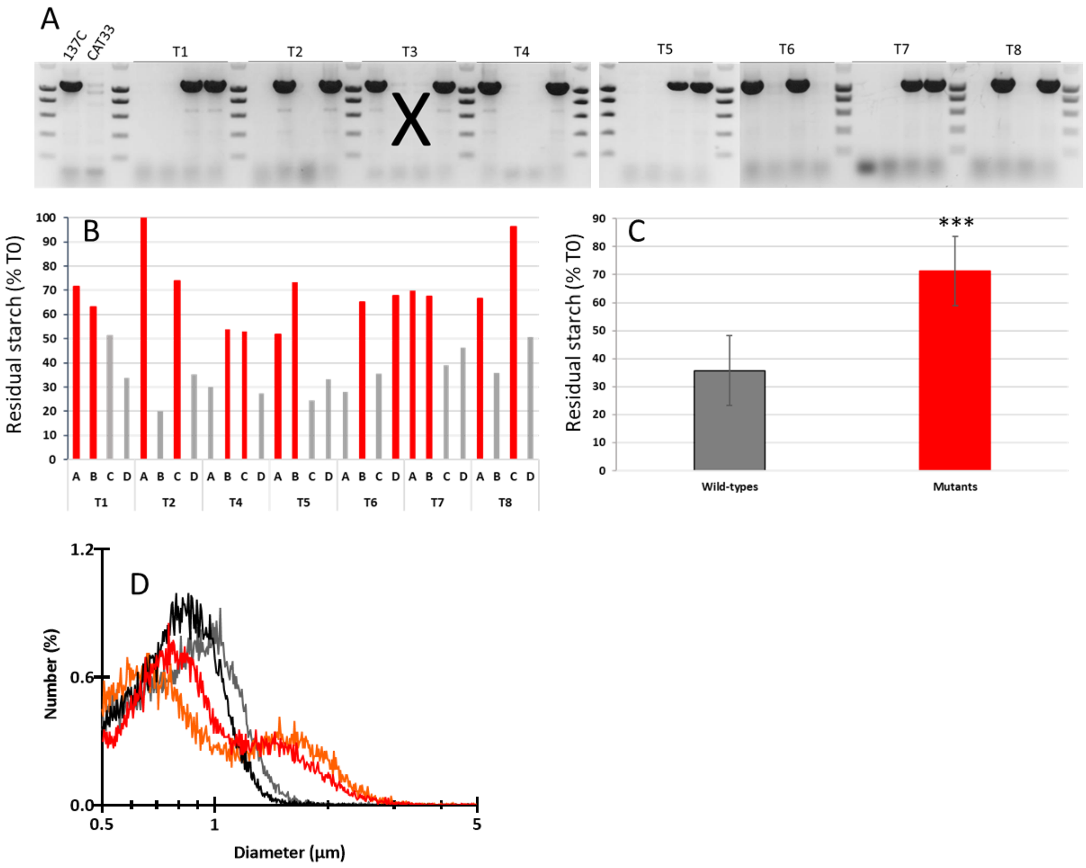

Supplementary figure S1 : Cosegregation analysis. (A) PCR analysis on the meiotic progeny arising from a cross between the *bsg1* mutant and the wild type strain 37. PCR analysis was performed on genomic DNA of each strain allowing the detection of a 1078 bp fragment revealing the integrity of the *Cre02.g091750* locus. Molecular weigth marker : Eurogentec Smartladder (B) Residual starch contents after 24h of degradation in the dark were determined for each strains arising from the cross between the *bsg1* mutant and the wild-type reference strain of opposite mating type 37. All the strains were cultivated during five days under nitrogen starvation. The cells were then transferred to TMP medium and incubated in the dark for 24 hours. Starch was assayed and is expressed as percentage of the initial amount. Results are displayed for the mutant (red columns) and the wild type progenies (grey columns) of the seven tetrads obtained from this cross. The name of each individual strain is indicated on the x-axis. Tetrad 3 was lost and was removed from the analysis. (C) The average starch amounts remaining after 24 hours of degradation in darkness in the whole wild-type and mutant progeny are indicated as means ± SE for the mutant (red column) and the wild-type progenies (grey column) *** P<0,001 by Student's *t* test. (D) The starch granule size distribution in the 4 strains composing one representative tetrad arising from the cross between the *bsg1-1* mutant and a wild-type strain is shown. These distributions were determined by analyzing 30,000 particles on a coulter counter. Starches were extracted for each strain grown 5 days under nitrogen starvation. The results are expressed in relative percentage (*y*-axis) of particles of a diameter ranging from 0.5 to 5 µm (*x*-axis, logarithmic scale). The two mutant progenies are indicated in red and orange while the two wild-type meiotic segregants are in grey and black.

Supplemental Table S1. Coiled-coil potential of BSG1 (*Cre02.g091750*).

The BSG1 amino acid sequence was subjected to analysis of coiled-coil potential using the web servers for each of the specified methods, under default running parameters. Strength of the predictions for a given coiled-coil sequence region were categorized as "Strong" when Threshold was > 90% with Marcoil (http://www.isrec.isb-sib.ch/webmarcoil/webmarcoilC1.html); P < 0.025 with PairCoil2 (http://groups.csail.mit.edu/cb/paircoil2/paircoil2.html); W = 21 and 28 P > 0.90 with PCOILS (http://toolkit.tuebingen.mpg.de/pcoils).

Marcoil	PairCoil2	PCOILS
Strong (303 to 328)<br>T=96.6%	Strong (302 to 384)<br>P<0.01	Strong (342 to 371)<br>P>0.906
Strong (331 to 367)<br>T= 99.9%		

Supplemental Table S2: Oligonucleotides used in this study. The annealing temperature and the expected amplification size products are indicated. Sequences corresponding to restriction enzyme sites are underlined. The experiments in which the primers were used are indicated in the last column, G : genotyping, C: cloning, R: RT-PCR

Locus	Primer name	Sequence	Size	Temperature	Experiment
Cre02.g091950	091950F	CCATTCCACTCTTCGCGTAGATC	700 bp	55°C	G
	091950R	CATGACGGCCTCCGGGTCAATGTCC			
Cre02.g091900	091900F	GTCGCATTAGTCCGTCACGAC	640 bp	60°C	G
	091900R	CGCTCTCAGCCGCAAGGCCTC			
Cre02.g091850	091850F	ACGGCGGTACCTGACATTAG	922 bp	58°C	G
	091850R	TGGGCAATCCAAACTGTGTA			
Cre02.g091750	091750F1	CCAGGGCACAGCCTGAACATGC	995 bp	53°C	G
	091750R1	CCACCTTCTTGTAGGAGTCATC			
	091750F2	GATGACTCCTACAAGAAGGTGG	912 bp	53°C	G
	091750R2	GATGTGAGCAACGCGCGCATG			
	091750F3	AAAGACCAGCTGTCAGACATGC	1048 bp	60°C	G
	091750R3	GCATGAACTCTGTCGGCGTGAG			
	091750F4	CTCACGCCGACAGAGTTCATGC	804 bp	60°C	G
	091750R4	ACCTCCACAGCCGCAGGATCTG			
	091750F5	TATCACATTGAACAGATCCTGC	926 bp	50°C	G
	091750R5	GCTTGACTAGTCTTACATAC			
	BSGF	CCGGGCGATGTTGACTCGGACAGC	1078 bp	62°C	G
	BSGR	ACAACTTCTGCCTGAGGCCTATCC			
	EcoRVFor	GATATCATGGGAGCCTGCCGGGTAGGTCG	3745 bp	60°C	C
	EcoRVRev	GATATCTTATTGCGCCTTCTCCTGTGATGC			
	RTF	GCAACTCTTCTGATGATGCTGGCGC	940 bp	60°C	R
	RTR	TATGCCACCACGCGGTTG			
PHOB	PhoBF	GCATGTTCCGCCAGACCA	736 bp	60°C	R
	PhoBR	TGCAGGAAGCGCCAGTTGA			
Cre02.g091700	091700F	GGCACCACAAATGCTAACC	849 bp	55°C	G
	091700R	GCGGCTTGTAAATCTTCTGG			

Original pictures for figure 1A

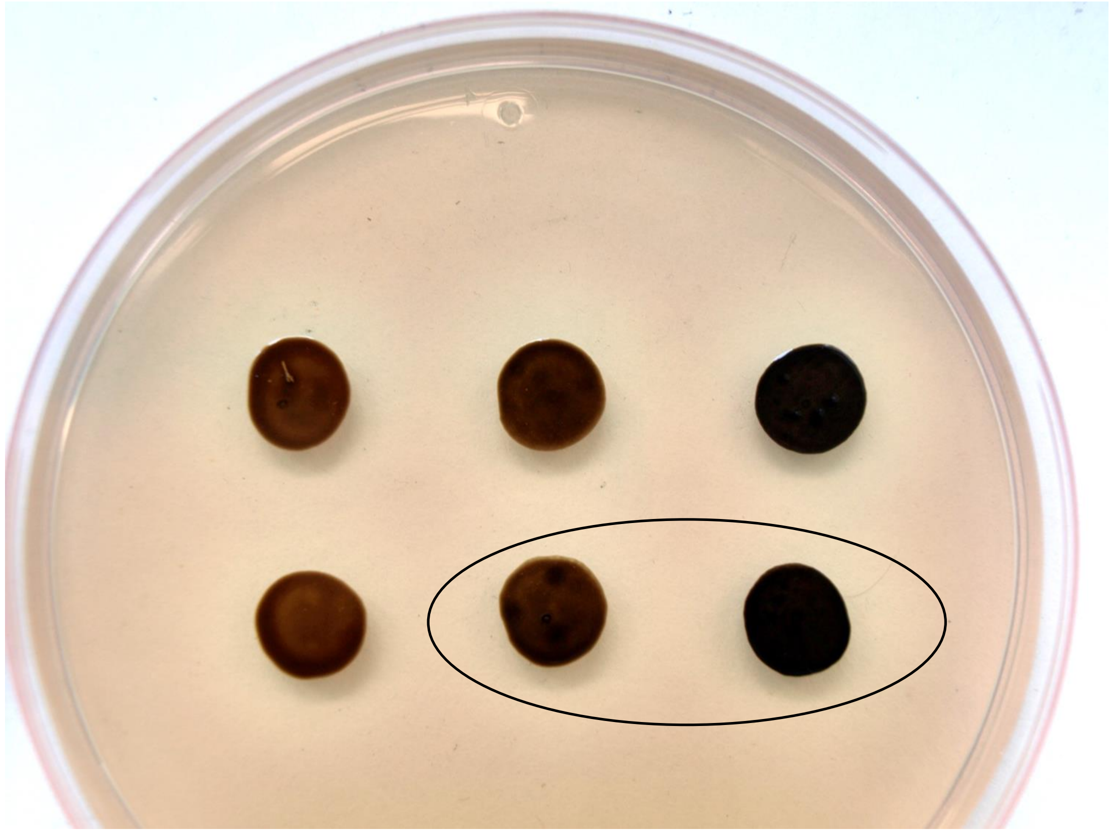

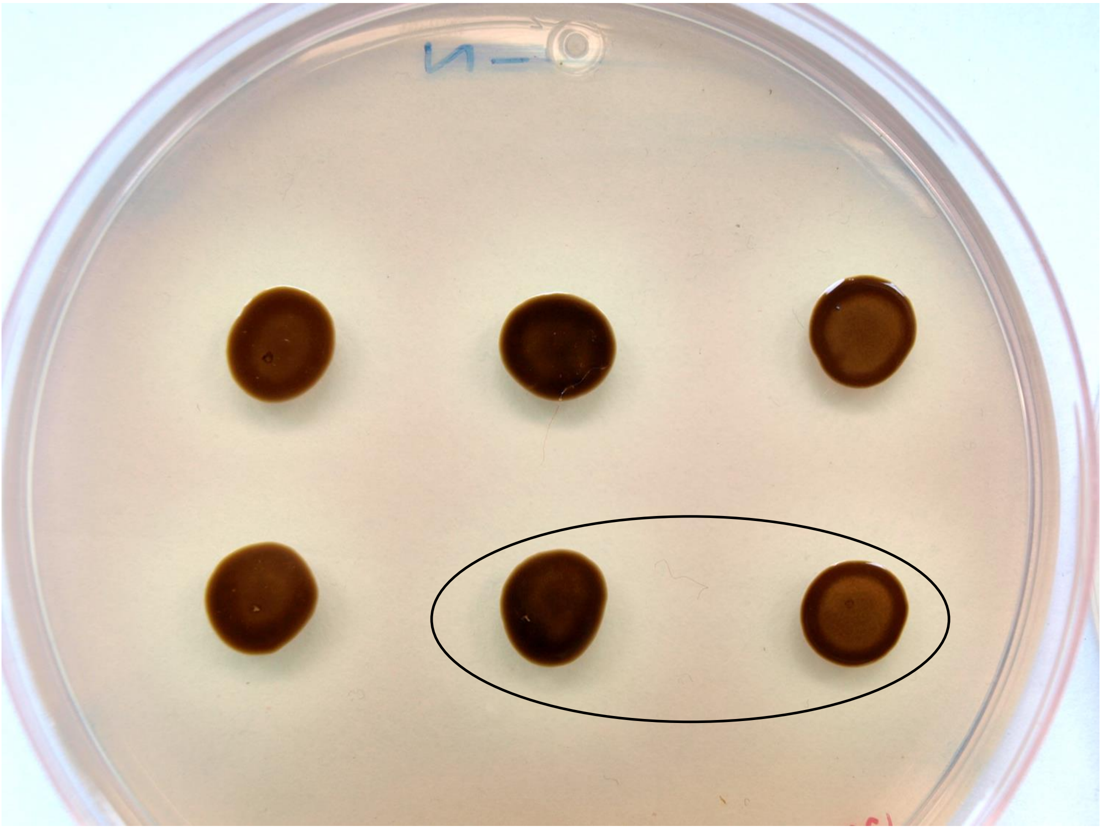

Original gels for figure 4A

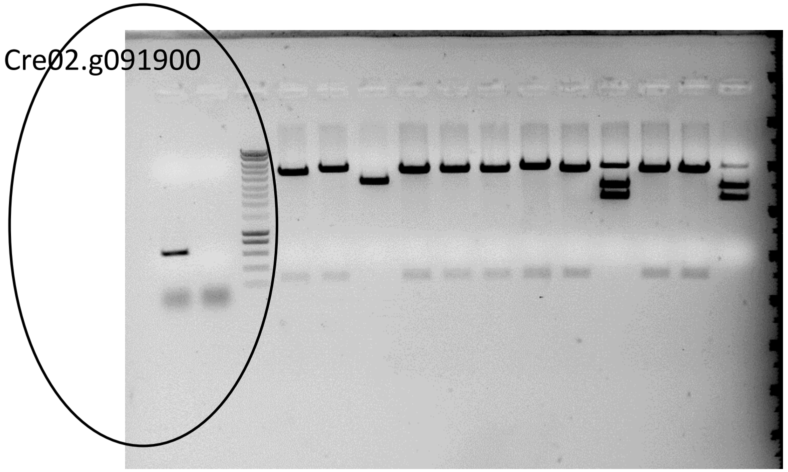

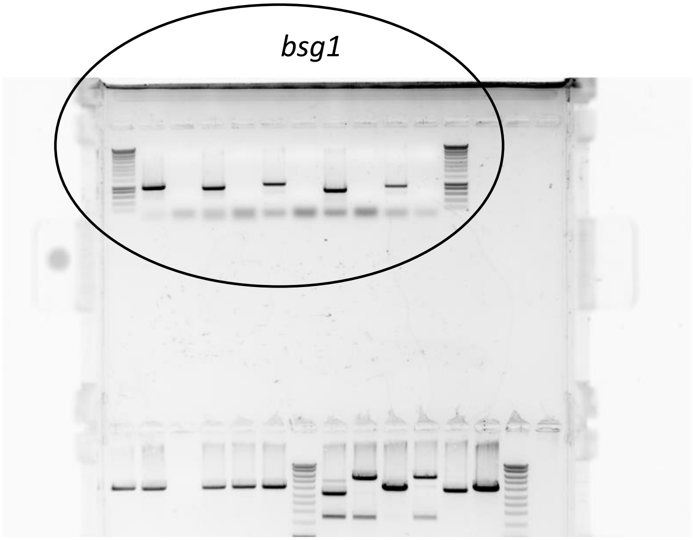

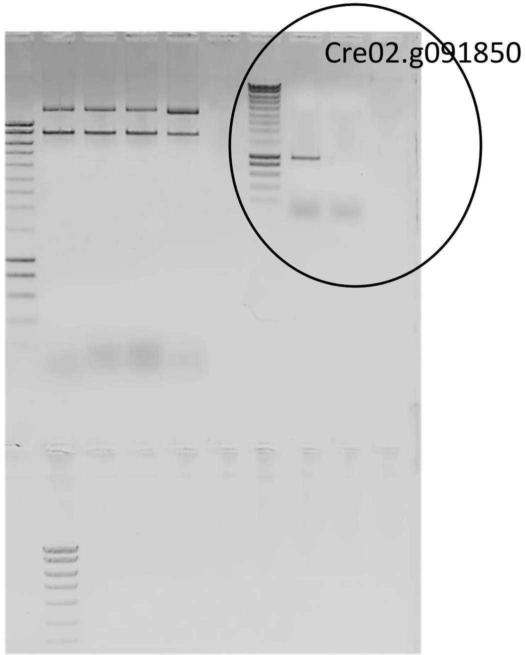

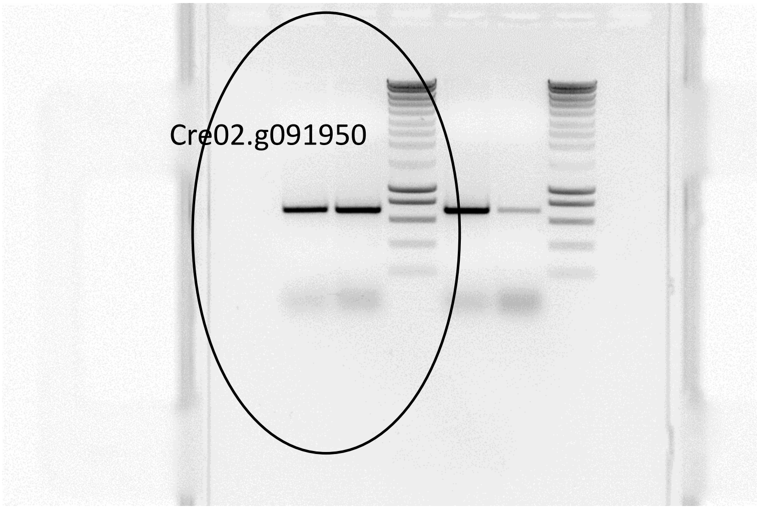

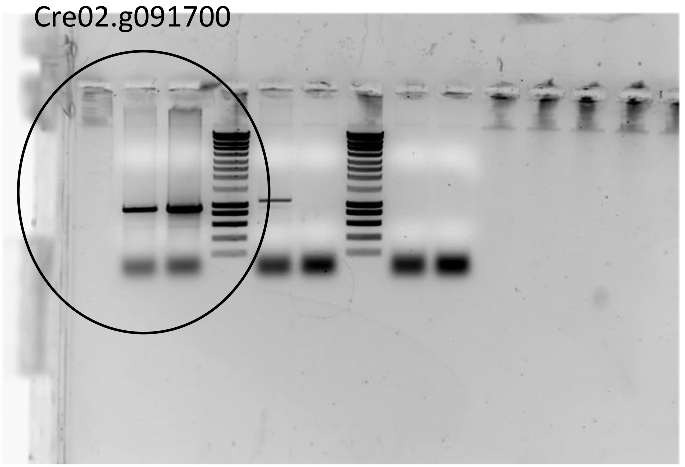

Original gels for figure 4B

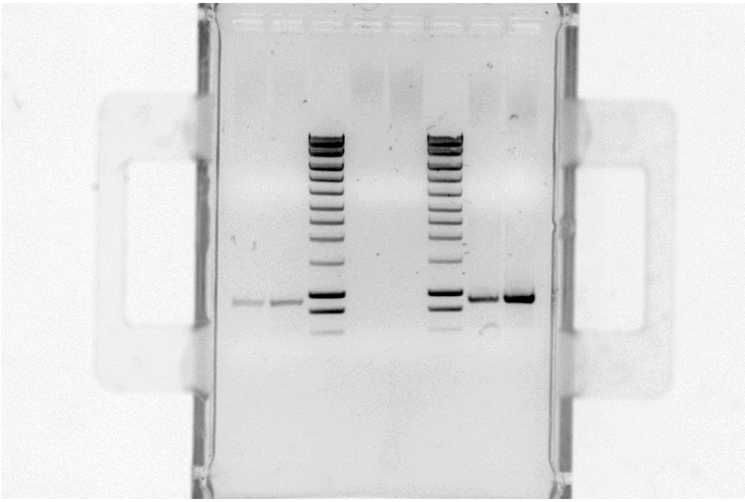

BSG1 RT-PCR

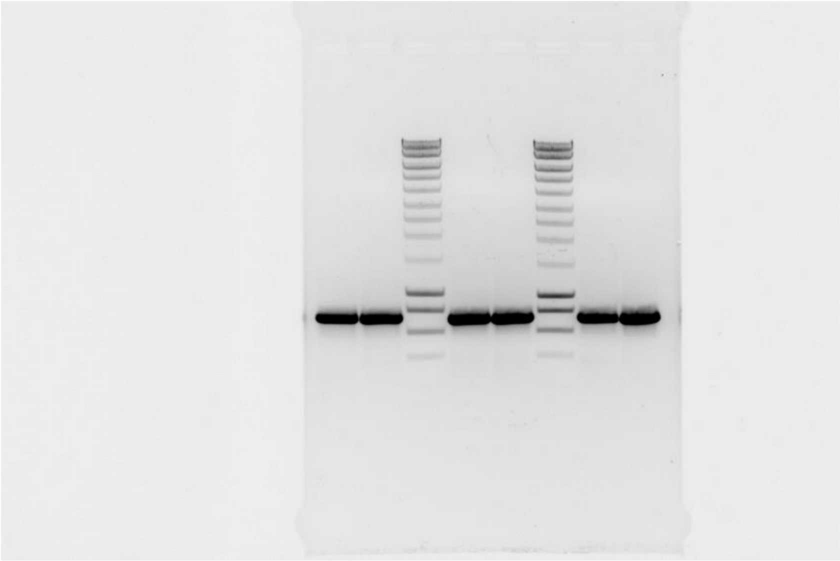

PHOB RT-PCR

Original gels for figure 4C

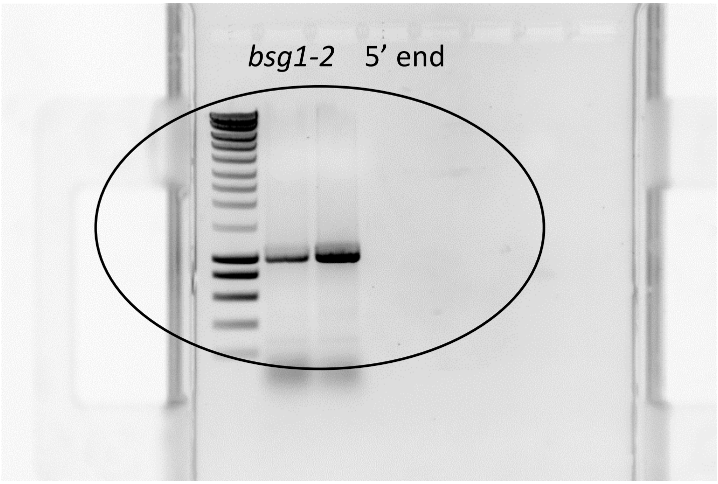

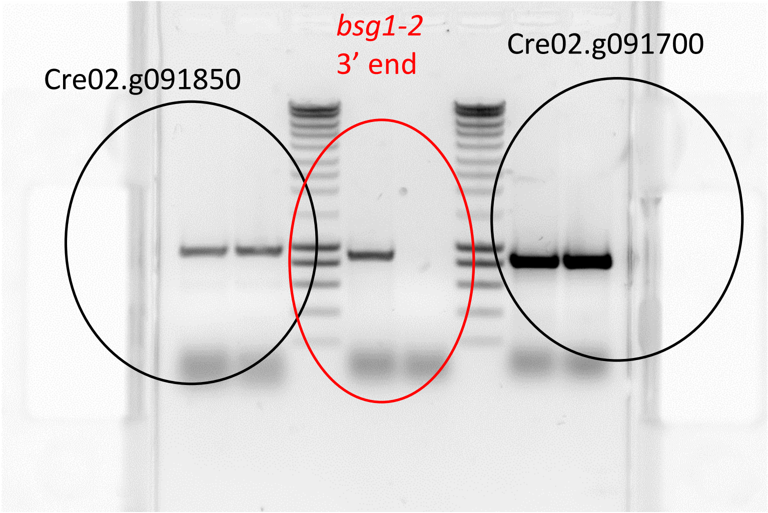

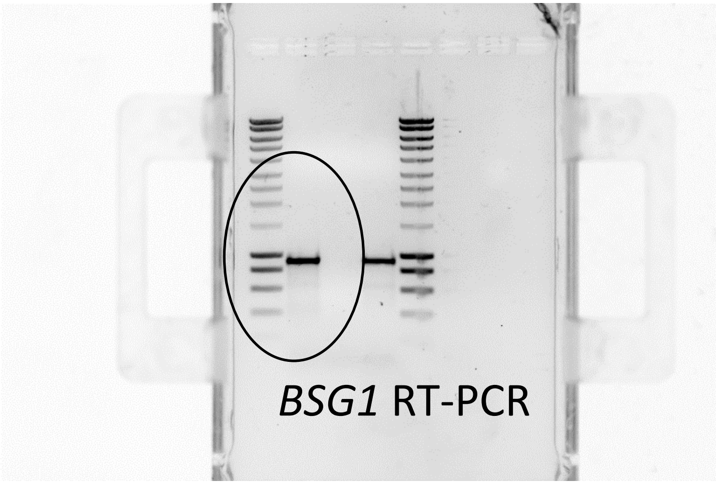

Original gels for figure 4D

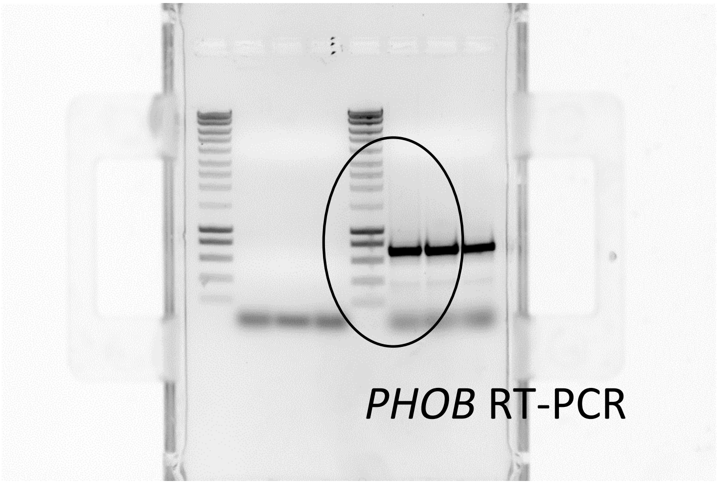

Supplement: Supplementary file 1 — Supplementary information [file 41598_2019_39506_MOESM1_ESM.pdf]
